# Intermediate catheter use is associated with intraprocedural rupture during coil embolization of ruptured intracranial aneurysms: a retrospective propensity score-matched study

**DOI:** 10.3389/fneur.2024.1401378

**Published:** 2024-07-12

**Authors:** Michiyasu Fuga, Toshihiro Ishibashi, Ken Aoki, Naoki Kato, Issei Kan, Shunsuke Hataoka, Gota Nagayama, Tohru Sano, Toshihide Tanaka, Yuichi Murayama

**Affiliations:** ^1^Department of Neurosurgery, The Jikei University School of Medicine, Tokyo, Japan; ^2^Department of Neurosurgery, The Jikei University School of Medicine, Katsushika Medical Center, Tokyo, Japan

**Keywords:** distal access catheter, intraoperative aneurysm rupture, intraoperative complication, intraoperative rupture, subarachnoid hemorrhage, balloon guiding catheter, endovascular treatment, hemorrhagic complication

## Abstract

**Introduction:**

An intermediate catheter (IMC) may pose a risk of intraprocedural rupture (IPR) during coil embolization of ruptured intracranial aneurysms (RIAs), because the pressure on the microcatheter and coil might be more direct. To verify this hypothesis, this study explored whether use of an IMC might correlate with an increased rate of IPR during coil embolization for RIAs.

**Methods:**

We retrospectively reviewed 195 consecutive aneurysms in 192 patients who underwent initial coil embolization for saccular RIAs at our institution between January 2007 and December 2023. Patients were divided into two groups with aneurysms treated either with an IMC (IMC group) or without an IMC (non-IMC group). To investigate whether IMC use increased the rate of IPR, a propensity score-matched analysis was employed to control for age, sex, maximal aneurysm size, neck size, bleb formation, aneurysm location, proximal vessel tortuosity, balloon-assisted coiling, type of microcatheter, and type of framing coil.

**Results:**

Ultimately, 43 (22%) coil embolization used IMC. In univariate analysis, the incidence of IPR was significantly higher in the IMC group compared with the non-IMC group (14.0 vs. 3.3%, *p* = 0.016). Propensity score matching was successful for pairs of 26 aneurysms in the IMC group and 52 aneurysms in the non-IMC group. The incidence of IPR was still significantly higher in the IMC group than in the non-IMC group (23.1 vs. 3.8%, *p* = 0.015). No significant differences in the incidences of ischemic complications and IMC-related parent artery dissection were observed between the two groups.

**Discussion:**

When using IMC for coil embolization of RIAs, the surgeons should be more careful and delicate in manipulating the microcatheter and inserting the coils to avoid IPR.

## Introduction

Following a report on the International Subarachnoid Aneurysm Trial, endovascular coiling of intracranial aneurysms has become more widespread as a less-invasive treatment associated with a higher independent survival rate compared with neurosurgical clipping (1). However, even with endovascular treatment, a risk of complications is inevitable. Intraprocedural rupture (IPR) is one of the most devastating and serious complications and represents a cause of poor outcome (2–4). Risk factors for IPR during coil embolization have been identified as small size aneurysm, ruptured intracranial aneurysms (RIAs), use of a microballoon, anterior or posterior communicating aneurysm, and irregularly shaped aneurysm (2, 4–28).

In recent years, along with advances and developments in endovascular devices, the intermediate catheter (IMC) has increasingly been employed for the coil embolization of intracranial aneurysms. The IMC might be delivered to more distal arteries, in turn making the microcatheter easier to control and thus facilitating high-density coil packing (29). On the other hand, support with an IMC may pose a risk of IPR during coil embolization of especially RIAs with fragile walls, because the pressure on the microcatheter and coil might be more direct. To verify this hypothesis, we investigated the association between use of an IMC and the incidence of IPR during coil embolization for RIAs.

## Materials and methods

### Study population

**Figure 1:**
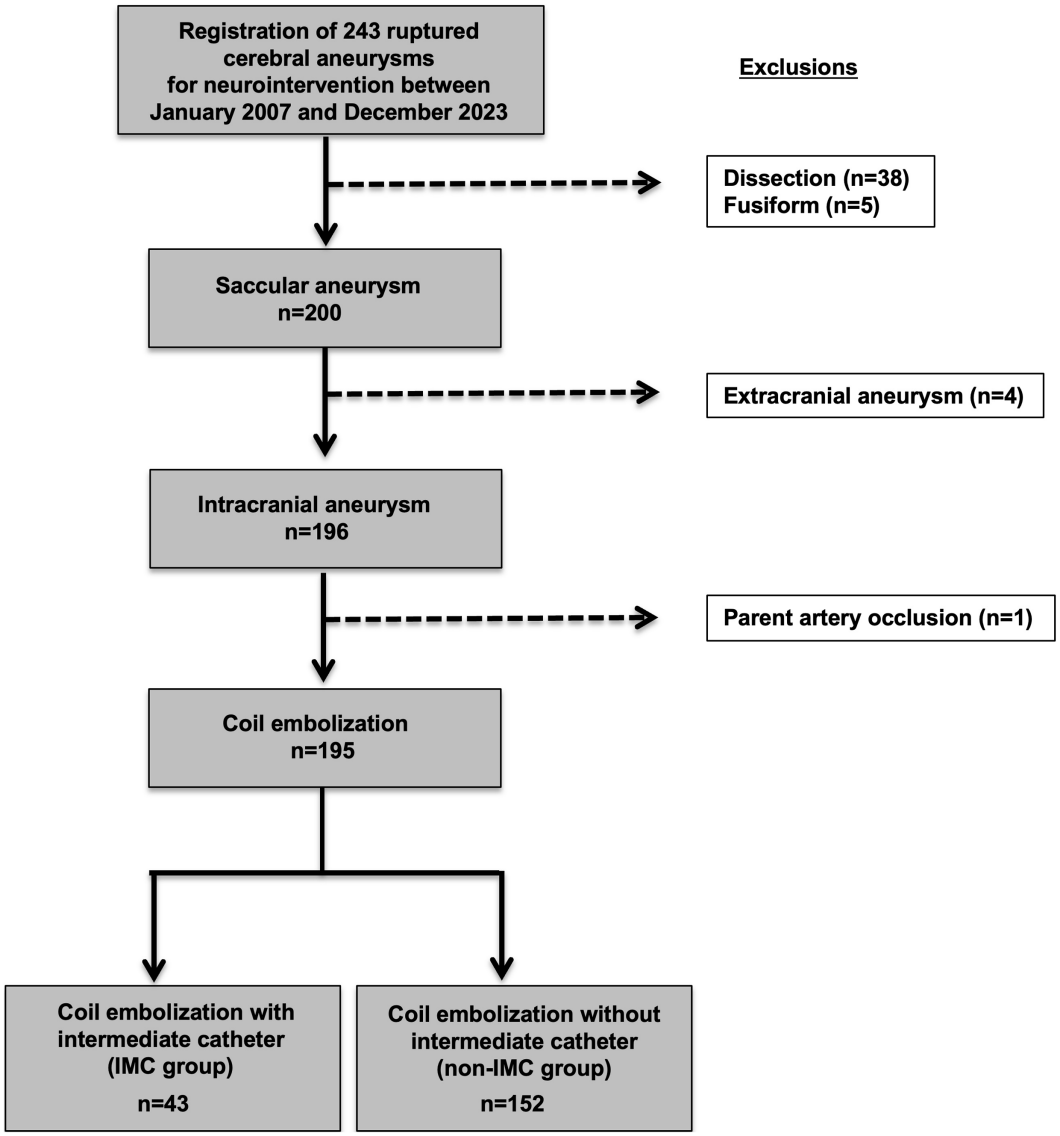
Flowchart for selection of saccular ruptured intracranial aneurysms for initial coil embolization and subsequent classification by use of IMC. A total of 243 consecutive initial endovascular treatments for ruptured cerebral aneurysms conducted at our institution between January 2007 and December 2023 were retrospectively reviewed. Patients with dissecting aneurysms (*n* = 38) and fusiform aneurysms (*n* = 5) were excluded. In addition, patients with extracranial aneurysm (*n* = 4) and aneurysms treated with parent artery occlusion (*n* = 1) were also excluded. Ultimately, 195 initial coil embolization of saccular ruptured intracranial aneurysms in 192 patients were included in the present study. Of the 195 saccular ruptured intracranial aneurysms, 43 patients were classified to the IMC group and 152 to the non-IMC group. IMC, Intermediate catheter.

A total of 243 consecutive initial endovascular treatments for ruptured cerebral aneurysms conducted at our institution between January 2007 and December 2023 were retrospectively reviewed. Patients with dissecting aneurysms (*n* = 38) and fusiform aneurysms (*n* = 5) were excluded. In addition, patients with extracranial aneurysm (*n* = 4) and aneurysms treated with parent artery occlusion (*n* = 1) were also excluded. Ultimately, 195 initial coil embolization of saccular RIAs in 192 patients were included in the present study (Figure 1). Patients were divided into two groups with aneurysms treated either with an IMC (IMC group) or without an IMC (non-IMC group).

### Data collection

The medical records and radiological data of these patients were retrospectively reviewed to obtain the following data: age, sex, medical history, family history of cerebral aneurysms, smoking and drinking histories, aneurysm characteristics, proximal vessel tortuosity, endovascular technique, and complications. A proximal vessel tortuosity was defined as two or more bends >90° before reaching the target lesion.

All aneurysms were evaluated for morphology and structure using rotational angiography with three-dimensional image reconstruction (Artis Q Biplane, Siemens Healthcare GmbH, Forchheim, Germany). Based on three-dimensional rotational angiographic images, aneurysm size was calculated using NeuroVision software (Cybernet Systems, Tokyo, Japan), which allows automatic measurement by only placing markers on the aneurysm and parent artery (30).

### Definitions of IPR and IMC

In accordance with previous reports (10, 13, 31), IPR was defined as a situation in which a microguidewire, microcatheter, or coil was displaced beyond the boundaries of the aneurysmal sac, with or without evident contrast extravasation on angiography.

An IMC was defined as a catheter inserted coaxially with a guiding catheter for the purpose of improving the maneuverability and stability of the microcatheter. The IMCs used included Tactics (Technorat Corporation, Aichi, Japan), Guidepost (Tokai Medical Products, Aichi, Japan), Cerulean (Medikit Co. Ltd., Tokyo, Japan), Sofia (MicroVention Terumo, Tustin, CA, United States), AXS Vecta (Stryker Neurovascular, Kalamazoo, MI, United States), DAC (Stryker Neurovascular), Navien (Medtronic, Irvine, CA, United States), and Asahi Fubuki (Asahi Intecc, Aichi, Japan).

### Endovascular treatment

All coil embolization procedures were accomplished under general anesthesia, in a standardized fashion, and were conducted exclusively by or under the supervision of certified interventional neurosurgeons. Coil embolization was performed without administration of antiplatelet therapy. After the first coil was inserted into the aneurysm, 4,000–5,000 U of heparin was given intravenously as a bolus infusion, followed by 1,000–2,000 U of heparin intermittently to maintain the activated clotting time during the procedure at a level at least twice the baseline value for that patient. Coil embolization was conducted as follows:

The femoral artery was selected as the access site, and for anterior circulation aneurysms, prior to 2010, a 6-8F guiding catheter or sheath without a balloon was placed in the cervical portion of the ICA, which was changed in preparation for IPR to place an 8Fr or 9Fr guiding catheter with a balloon beginning in 2010. For posterior circulation aneurysms, on the other hand, a 5-8F guiding catheter or sheath without a balloon was directed into the V1 (the pre-foraminal segment and ranging from the origin of the VA to the transverse foramen of the sixth cervical vertebra) or V2 (the foraminal segment and ranging from the transverse foramen of the sixth cervical vertebra to the transverse foramen of the second cervical vertebra) segment of the VA. The decision to use IMC was left to the discretion of the surgeon. A 0.0165 or 0.017-in microcatheter was navigated into the aneurysm over a 0.014-in microguidewire. The type of coils used was left to the discretion of the surgeon. In cases of wide-necked aneurysms (neck size >4 mm or dome-to-neck ratio < 2), balloon-assisted, double-catheter, or stent-assisted technique was applied, otherwise primary coiling was employed. In our country, coil embolization using the stent-assisted technique for RIAs has not been covered by national insurance, so it was placed only when no other procedure was available and after obtaining full explanation and consent from the patient or his/her family.

### Complications

Complications associated with the procedure were categorized as ischemic, hemorrhagic, or IMC-related parent artery dissection, with IPR assigned as a hemorrhagic complication. Symptomatic complications were defined as an increase of ≥1 in mRS score compared with the preoperative level. This study adhered to the Declaration of Helsinki. Our institutional review board waived the need for informed consent due to the retrospective design.

### Statistical analyses

For the comparison of baseline characteristics between IMC and non-IMC groups, the Mann–Whitney U test and Fisher’s exact test were applied to continuous variables and categorical variables, respectively. A 1:2 propensity score matching between IMC and non-IMC groups was performed using the nearest neighbor method, without replacement and with a caliper width of 0.20, to match groups on covariates of age, sex, proximal vessel tortuosity, type of microcatheter, type of framing coil, and previously reported risk factors for IPR, including maximal aneurysm size, neck size, bleb formation, aneurysm location, and balloon-assisted coiling (2, 4–28). All statistical analyses were performed using R and R Commander-based Easy R (EZR) software (Saitama Medical Center, Jichi Medical School, Saitama, Japan) (32). Values of *p* < 0.05 were considered significant.

## Results

### Clinical, anatomic, and procedural characteristics

Among the 195 RIAs, 43 (22%) underwent coil embolization using an IMC. Clinical, anatomic, and procedural characteristics are shown in Table 1. No significant differences in age, sex, medical history, family history of cerebral aneurysms, and smoking history were apparent between groups. Patients in the IMC group were significantly more likely to have a history of drinking than those in the non-IMC group (*p* = 0.026).

**Table 1:** Clinical, anatomic, and procedural characteristics of saccular ruptured intracranial aneurysms before and after propensity score matching between IMC and non-IMC groups.

Characteristics	Total population			Propensity score matching^§^		
	IMC (*n* = 43)	Non-IMC (*n* = 152)	*p* value	IMC (*n* = 26)	Non-IMC (*n* = 52)	*p* value
Age, years	55 [48, 69]	55 [46, 67]	0.92	53 [45, 62]	56 [49, 64]	0.30
Sex, female	23 (53.5)	88 (57.9)	0.61	13 (50.0)	28 (53.8)	0.81
Medical history						
Hypertension	15 (34.9)	56 (36.8)	0.86	10 (38.5)	24 (46.2)	0.63
Diabetes mellitus	3 (7.0)	5 (3.3)	0.38	2 (7.7)	2 (3.8)	0.60
Dyslipidemia	4 (9.3)	14 (9.2)	1	2 (7.7)	9 (17.3)	0.32
Prior stroke	1 (2.3)	8 (5.3)	0.69	0 (0)	6 (11.5)	0.17
Polycystic kidney	0 (0)	1 (0.7)	1	0 (0)	1 (1.9)	1
Family history of cerebral aneurysms	3 (7.0)	9 (5.9)	0.73	2 (7.7)	2 (3.8)	0.60
Smoking						
Current	9 (20.9)	16 (10.5)	0.17	6 (23.1)	3 (5.8)	0.068
Past	5 (11.6)	16 (10.5)		2 (7.7)	9 (17.3)	
None	29 (67.4)	120 (78.9)		18 (69.2)	40 (76.9)	
Drinking	11 (25.6)	17 (11.2)	0.026^*^	8 (30.8)	7 (13.5)	0.13
Aneurysm characteristic						
Maximal aneurysm size, mm	4.1 [3.3, 5.5]	5.5 [4.1, 6.8]	<0.001^*^	4.6 [3.6, 6.3]	4.6 [3.7, 5.6]	0.80
Neck size, mm	2.9 [2.0, 3.7]	3.4 [2.6, 4.7]	0.006^*^	3.3 [2.6, 3.7]	3.0 [2.3, 3.9]	0.59
Bleb formation	25 (58.1)	122 (80.3)	0.005^*^	17 (65.4)	40 (76.9)	0.29
Aneurysm location						
ACA/ACoA	27 (62.8)	49 (32.2)	0.002^*^	13 (50.0)	26 (50.0)	1
MCA	3 (7.0)	14 (9.2)		2 (7.7)	3 (5.8)	
ICA	12 (27.9)	67 (44.1)		10 (38.5)	20 (38.5)	
Posterior circulation	1 (2.3)	22 (15)		1 (3.8)	3 (5.8)	
Anterior communicating aneurysm	22 (51.2)	47 (30.9)	0.019^*^	12 (46.2)	26 (50.0)	0.81
Posterior communicating aneurysm	8 (18.6)	48 (31.6)	0.13	7 (26.9)	12 (23.1)	0.78
Proximal vessel tortuosity	7 (16.3)	31 (20.4)	0.67	3 (11.5)	9 (17.3)	0.74
Endovascular technique						
Primary coiling	30 (69.8)	88 (59.5)	0.76	15 (57.7)	30 (57.7)	0.78
Balloon-assisted	2 (4.7)	10 (6.8)		2 (7.7)	3 (5.8)	
Double-catheter	10 (23.3)	45 (30.4)		9 (34.6)	16 (30.8)	
Stent-assisted	1 (2.3)	5 (3.4)		0 (0)	3 (5.8)	
Without balloon	41 (95.3)	138 (93.2)	1	24 (92.3)	49 (94.2)	1
Type of microcatheter						
Excelsior SL-10^†^	41 (95.3)	149 (98.0)	0.31	26 (100)	51 (98.1)	1
Other microcatheters^††^	2 (4.7)	3 (2.0)		0 (0)	1 (1.9)	
Type of framing coil						
Target^†^	36 (83.7)	68 (44.7)	<0.001^*^	19 (73.1)	39 (75.0)	0.11
Matrix2^†^	1 (2.3)	56 (36.8)		1 (3.8)	8 (15.4)	
GDC^†^	2 (4.7)	27 (17.8)		2 (7.7)	5 (9.6)	
Other coils^†††^	4 (9.3)	1 (0.7)		4 (15.4)	1 (1.9)	
Non-Target coil	7 (16.3)	84 (55.3)	<0.001^*^	7 (26.9)	13 (25.0)	1
Complication						
Ischemic	1 (2.3)	4 (2.6)	1	1 (3.8)	1 (1.9)	1
Symptomatic^††††^	1 (2.3)	3 (2.0)	1	1 (3.8)	1 (1.9)	1
Hemorrhagic	6 (14.0)	5 (3.3)	0.016^*^	6 (23.1)	2 (3.8)	0.015^*^
Symptomatic^††††^	1 (2.3)	2 (1.3)	0.53	1 (3.8)	1 (1.9)	1
IPR	6 (14.0)	5 (3.3)	0.016^*^	6 (23.1)	2 (3.8)	0.015^*^
IMC-related parent artery dissection	0 (0)	0 (0)	NA	0 (0)	0 (0)	NA

ACA, Anterior cerebral artery; ACoA, Anterior communicating artery; BA, Basilar artery; GDC, Guglielmi detachable coil; ICA, Internal carotid artery; IMC, Intermediate catheter; IPR, Intra-procedural rupture; MCA, Middle cerebral artery; NA, Not available; PCA, Posterior cerebral artery; PICA, Posterior inferior cerebellar artery; SCA, Superior cerebellar artery; VA, Vertebral artery. ^†^Stryker Neurovascular, Kalamazoo, MI, United States. ^††^Excelsior 1,018 (Stryker Neurovascular), Phenom 17 (Medtronic, Irvine, CA, United States), Headway 17 (MicroVention Terumo, Tustin, CA, United States), and Headway Duo (MicroVention Terumo) are included. ^†††^ED Coil (Kaneka, Osaka, Japan), Axium (Medtronic), and Optima (Balt, Montmorency, France) are included. ^††††^Symptomatic complications were defined as an increase of one or more points in mRS score compared with the preoperative level. ^*^p < 0.05. Unless otherwise indicated, values represent the number of aneurysms (%) or median and interquartile range. Not all percentages total 100% due to rounding. ^§^After 1:2 matching by propensity score matching, controlling for age, sex, maximal aneurysm size, neck size, bleb formation, aneurysm location, proximal vessel tortuosity, balloon-assisted coiling, type of microcatheter, and type of framing coil.

Compared with the non-IMC group, the IMC group had significantly smaller aneurysm size (4.1 [IQR: 3.3, 5.5] mm vs. 5.5 [IQR: 4.1, 6.8] mm, *p* < 0.001) and neck size (2.9 [IQR: 2.0, 3.7] mm vs. 3.4 [IQR: 2.6, 4.7] mm, *p* = 0.006) and a lower frequency of bleb formation (58.1 vs. 80.3%, *p* = 0.005). Regarding aneurysm location, use of an IMC was significantly more frequent in the treatment of anterior communicating artery (ACoA) aneurysms (51.2 vs. 30.9%, *p* = 0.019). The rate of proximal vessel tortuosity, endovascular technique, and type of microcatheter were not significantly different between the two groups. Regarding the type of framing coil, the rate of target coil (Stryker Neurovascular) use was significantly higher in the IMC group compared with the non-IMC group (*p* < 0.001). Rates of ischemic complications and IMC-related parent artery dissection did not differ significantly between groups. Hemorrhagic complications were all IPR, and the incidence of IPR was significantly higher in the IMC group than in the non-IMC group (14.0 vs. 3.3%, *p* = 0.016) (Table 1).

### Characteristics of IPR in the IMC group

Intraprocedural ruptures with IMC in RIAs are presented in Table 2. Of the six patients who developed IPR, median age was 53 years (IQR, 34–58 years), with a predominance of female patients (*n* = 4, 66.7%). Aneurysm location was the ACoA in five cases (83.3%) and posterior communicating artery in 1 (16.7%). Median maximal aneurysm size was 4.1 mm (IQR, 3.2–4.5 mm), and bleb formation was observed in all aneurysms. All microcatheters used for endovascular treatment were Excelsior SL-10 (Stryker Neurovascular). The IMC used for endovascular treatment was a 6-Fr Sofia in three patients (50%) and a 4-Fr Cerulean, a 3.2-Fr Guidepost, and a 3.2-Fr Tactics in one patient each (16.7%). The location of the IMC used for coil embolization was the ICA cavernous segment in three patients (50%), the ICA supraclinoid segment in two patients (33.3%), and the pre-communicating segments (A1) of the anterior cerebral artery in one patient (16.7%), with the left side predominating (66.7%), and three IMCs (50%) placed intradurally. The endovascular technique were double-catheter and primary coiling in three patients (50%) each. Causes of IPR were coils in five patients (83.3%) and microcatheter in one patient (16.7%), with IPR occurring most frequently at the time of insertion of the framing coil (66.7%). The types of perforated framing coils included three cases of target coils and one case of optima coil (Balt, Montmorency, France), with a variety of coil stiffness, and coil sizes smaller than the maximum aneurysm diameter were selected for all aneurysms. The mRS score 1 year after surgery was 0 for all patients.

**Table 2:** Characteristics of IPR in the IMC group.

Case	Age (years), sex	Aneurysm location	Maximal aneurysm size, mm	Bleb formation	Types of microcatheter	Types of IMC	Intermediate catheter size, Fr	Location of IMC, side	Endovascular technique	Causes of IPR	Perforated framing coil	Prognosis 1 year after surgery, mRS score
1	58/M	ACoA	3.2	Yes	Excelsior SL-10	Cerulean	4	ICA cavernous segment/right	Primary coiling	Microcatheter	NA	0
2	56/M	ACoA	3.6	Yes	Excelsior SL-10	Tactics	3.2	ICA supraclinoid segment/left	Primary coiling	Coil (filling coil)	NA	0
3	33/F	ACoA	4.5	Yes	Excelsior SL-10	Sofia	6	ICA supraclinoid segment/left	Double-catheter	Coil (framing coil)	Target 360 ultrasoft 3 mm × 6 cm	0
4	49/F	ACoA	6.5	Yes	Excelsior SL-10	Sofia	6	ICA cavernous segment/left	Double-catheter	Coil (framing coil)	Target 360 soft 3.5 mm × 10 cm	0
5	83/F	PCoA	4.5	Yes	Excelsior SL-10	Sofia	6	ICA cavernous segment/right	Double-catheter	Coil (framing coil)	Target 3D standard 4 mm × 8 cm	0
6	34/F	ACoA	1.5	Yes	Excelsior SL-10	Guidepost	3.2	A1 segment/left	Primary coiling	Coil (framing coil)	Optima Complex Supersoft 1 mm × 2 cm	0

M, Male; F, Female; ACoA, Anterior communicating artery; A1, Pre-communicating segments of the anterior cerebral artery; ICA, Internal carotid artery; IMC, Intermediate catheter; IPR, Intra-procedural rupture; mRS = Modified Ranking scale; NA, Not available; and PCoA, Posterior communicating artery.

### Association between IMC use and IPR after propensity score matching

Propensity score matching was successful for pairs of 26 aneurysms in the IMC group and 52 aneurysms in the non-IMC group (Table 1). After matching for age, sex, maximal aneurysm size, neck size, bleb formation, aneurysm location, proximal vessel tortuosity, balloon-assisted coiling, type of microcatheter, and type of framing coil, the incidence of IPR in the IMC and non-IMC groups was compared. The incidence of IPR was still significantly higher in the IMC group than in the non-IMC group (23.1 vs. 3.8%, *p* = 0.015) (Table 1). There were no significant differences between the two groups in the incidence of ischemic complications and IMC-related parent artery dissection.

### Illustrative case

**Figure 2:**
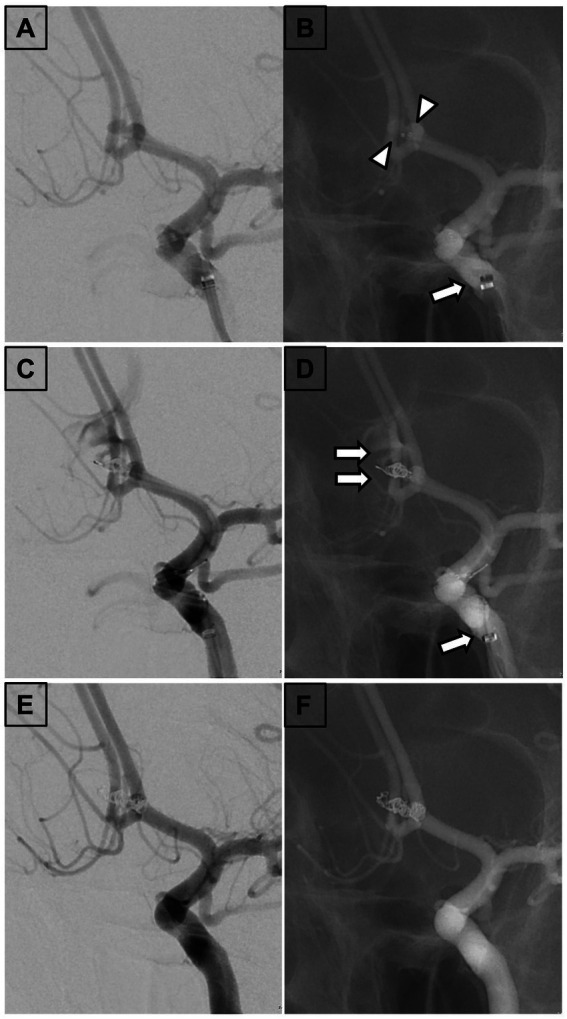
Findings in a 33-year-old woman. **(A)** Left internal carotid arteriography. A 33-year-old woman presenting with severe headache revealed a ruptured anterior communicating artery aneurysm with a maximum diameter of 4.5 mm. **(B)** Fluoroscopic view demonstrating aneurysm embolization under a double-catheter technique with a 6-Fr Sophia catheter guided as an IMC to the supraclinoid segment of the left internal carotid artery. **(C)** Left internal carotid arteriography revealing perforation of the aneurysm sac during insertion of a framing coil into the aneurysm and extravasation of contrast medium. **(D)** Fluoroscopic view showing coil displacement beyond the boundaries of the aneurysmal sac. **(E)** Left internal carotid arteriography demonstrating that the coil was immediately filled into the aneurysm from the other unperforated catheter. The perforated coil was inserted in a dumbbell fashion from outside to inside the aneurysm to seal the perforation and stop the bleeding. **(F)** Fluoroscopic view indicating insertion of the perforated coil in a dumbbell fashion from outside to inside the aneurysm to seal the perforation. IMC, Intermediate catheter; Arrowhead, Tip of the microcatheter; Arrow, Tip of the IMC; and Double arrow, Perforated coil loop.

A 33-year-old woman without previous medical history presented with severe headache and was transported to the emergency department at our institution. CT and DSA of the brain showed SAH due to ruptured ACoA aneurysm with a maximum diameter of 4.5 mm (Figure 2A). Subsequently, the aneurysm was embolized with coil under the double-catheter technique with a 6-Fr Sofia catheter guided as an IMC to the left ICA supraclinoid segment (Figure 2B). During insertion of a framing coil into the aneurysm, the sac of the aneurysm was perforated, and angiography revealed extravasation of contrast medium (Figure 2C). The coil was displaced beyond the boundaries of the aneurysmal sac (Figure 2D). For hemostatic purposes, coils were immediately packed into the aneurysm from the other unperforated catheter. After confirming that the contrast agent had stopped leaking, the perforated coil was inserted in a dumb-bell fashion from outside the aneurysm to inside the aneurysm, sealing the perforated portion (Figures 2E,F). Post-treatment CT scan demonstrated retention of contrast medium in the subarachnoid space.

## Discussion

The present study revealed that the use of an IMC was significantly associated with the incidence of IPR in coil embolization of RIAs. RIAs have previously been described as a risk factor for IPR (2, 10, 33). A meta-analysis by Cloft et al. demonstrated that the risk of IPR during coil embolization was significantly higher in RIAs than in unruptured intracranial aneurysms (UIAs) (4.1 vs. 0.5%, *p* < 0.001) (2). IPR of an UIA requires a new tear in the aneurysm wall, whereas IPR of a RIA can occur either by dislodging a clot that has occluded the original rupture point or by further tearing of the already torn and fragile aneurysm wall. RIAs may have more fragile walls than UIAs and thus require more delicate microcatheter manipulation and coil insertion than UIAs.

### IMC and IPR in RIAs

According to a previous literature review, the overall incidence of IPR was 4.47% (393/8791) for RIAs (25). In the present study, the incidence of IPR without IMC in RIAs was 3.3%, which is comparable to the previous meta-analysis. On the other hand, the incidence of IPR with IMC was as high as 14.0%. Causes of IPR have been previously described as microguidewires, coils, and microcatheters (8). The present study revealed coils as the most frequent cause of IPR in coil embolization with IMC for RIAs. Furthermore, the majority of IPRs occurred during first coil insertion in the framing phase. In the present study, the type of framing coil may not affect the incidence of IPR because the rate of IPR in the IMC group was not significantly different from that in the non-IMC group even after adjusting for the two groups in a propensity score-matched analysis. One possible mechanism for the increased risk of rupture with IMC could be that the support by the IMC may have increased the more direct pressure against the microcatheter and coil, which in turn could have increased the more direct pressure against the aneurysm wall. Such phenomena could explain the higher incidence of IPR with IMC use, particularly during framing coil insertion. In RIAs, if an IMC is employed, the coils should be inserted gently and with care, particularly during insertion of the first coil in the framing phase.

In the IMC group as indicated in Table 2, despite IPR, the prognosis 1 year after surgery was favorable with mRS 0 in all patients. The reason for the favorable prognosis may be attributed to the use of balloon guiding catheters for the treatment of RIAs, as our research group previously demonstrated (28). This means that even if the aneurysm ruptured intraoperatively, the balloon dilation enabled rapid hemostasis. Therefore, the rupture point could be treated while stopping the bleeding, which led to a favorable prognosis. As was shown in the results of the present study, the use of IMC in coil embolization for RIAs can increase the risk of IPR. When using IMC, employing balloon guiding catheters may not worsen the patient’s prognosis because even if IPR occurs, rapid hemostasis can be achieved by balloon dilation.

### ACoA aneurysms and IMC

In the present study, an IMC was significantly more frequently used for the coil embolization of ACoA aneurysms. This is because aneurysms at this site are distal and along a tortuous path, and navigation with microcatheters is often unstable and technically challenging (34). IMC can contribute to an increased volume embolization ratio by improving the maneuverability and stability of microcatheters (29). However, ACoA aneurysms have previously been alerted as a risk factor for IPR (22, 23). Kawabata et al. performed coil embolization in 1375 patients (1,406 UIAs), and IPR occurred in 20 aneurysms of 20 patients (1.4%). Multivariate analyses revealed that ACoA aneurysms were independently associated with IPR [OR: 7.54 (95% CI: 2.82–19.30), *p* = 0.0001] (23). In fact, the present study showed that IPR occurred in 5 of 22 (22.7%) ruptured ACoA aneurysms in coil embolization with an IMC. Based on the above, when the use of IMC for coil embolization of ruptured ACoA aneurysms, surgeons should keep in mind that more careful microcatheter manipulation and coil insertion may be necessary to avoid the IPR.

## Limitations

The present study has several limitations that should be noted when interpreting the findings. First, the long observation period may have allowed for improvements in endovascular device during that time. Newer coils, microguidewires, and microcatheters may reduce the risk of IPR. However, in the present study, the two groups were compared after propensity score matching with respect to the type of microcatheter and the type of framing coil, both of which are potential confounders of IPR. In addition, more maneuverable IMCs have only recently been developed (35–39). Nevertheless, the incidence of IPR was significantly increased in the coil embolization of RIAs with IMC compared with those without IMC. Therefore, regardless of improvements in endovascular device, IMC can increase the risk of IPR.

Second, this was a retrospective, single-center study, so multi-center and prospective studies should be conducted in the future to verify our findings. Despite these limitations, the present study showed that use of an IMC was significantly associated with IPR in the coil embolization of RIAs.

## Conclusion

When using IMC for coil embolization of RIAs, especially in ACoA aneurysms, the surgeons should be more careful and delicate in manipulating the microcatheter and inserting the coils to avoid IPR.

## Data availability statement

The raw data supporting the conclusions of this article will be made available by the authors, without undue reservation.

## Ethics statement

The studies involving humans were approved by the Ethics Committee of the Jikei University School of Medicine. The studies were conducted in accordance with the local legislation and institutional requirements. The ethics committee/institutional review board waived the requirement of written informed consent for participation from the participants or the participants' legal guardians/next of kin due to the retrospective nature of the study.

## Author contributions

MF: Conceptualization, Data curation, Formal Analysis, Investigation, Methodology, Writing – original draft, Writing – review & editing. TI: Conceptualization, Data curation, Methodology, Supervision, Validation, Writing – review & editing. KA: Data curation, Investigation, Methodology, Supervision, Validation, Writing – review & editing. NK: Data curation, Formal Analysis, Investigation, Project administration, Validation, Visualization, Writing – review & editing. IK: Conceptualization, Data curation, Resources, Supervision, Validation, Visualization, Writing – review & editing. SH: Investigation, Methodology, Project administration, Supervision, Writing – review & editing. GN: Investigation, Methodology, Validation, Visualization, Writing – review & editing. TS: Formal Analysis, Investigation, Methodology, Validation, Writing – review & editing. TT: Conceptualization, Data curation, Formal Analysis, Investigation, Methodology, Project administration, Supervision, Validation, Visualization, Writing – review & editing. YM: Validation, Writing – review & editing, Conceptualization, Investigation, Methodology, Supervision.
